# Network-Based Differential Analysis to Identify Molecular Features of Tumorigenesis for Esophageal Squamous Carcinoma

**DOI:** 10.3390/molecules23010088

**Published:** 2018-01-01

**Authors:** Suxia Jiang, Qi Zhang, Yansen Su, Linqiang Pan

**Affiliations:** 1 School of Electrical and Information Engineering, Zhengzhou University of Light Industry, Zhengzhou 450002, China; jiangsx913@126.com (S.J.); issacharzhang@yeah.net (Q.Z.); 2 Institute of Bio-inspired Intelligence and Mining Knowledge, School of Computer Science and Technology, Anhui University, Hefei 230039, China; 3 Key Laboratory of Image Processing and Intelligent Control, School of Automation, Huazhong University of Science and Technology, Wuhan 430074, China

**Keywords:** esophageal squamous carcinoma, gene, biomarker, centrality measure

## Abstract

Esophageal cancer has a poor prognosis and high mortality rate across the world. The diagnosis and treatment of esophageal cancer are hindered by the limited knowledge about the pathogenesis mechanisms of esophageal cancer. Esophageal cancer has two major subtypes, squamous and adenocarcinoma. In this work, we proposed a method to select candidate biomarkers of esophageal squamous carcinoma based on the topological differential analysis between the gene–gene interaction networks for esophageal squamous carcinoma and normal cells. We established the gene–gene interaction networks for esophageal squamous carcinoma and normal based on the correlation of genes. For each gene, we firstly calculated and compared five centrality measures, which could reflect the topological property of a network. According to five centrality measures, the genes with large differences between the two networks were regarded as candidate biomarkers for esophageal squamous carcinoma. A total of 21 candidate biomarkers were identified for esophageal squamous carcinoma, and seven of them have been confirmed to be biomarkers of esophageal-12 squamous carcinoma by previous research. In addition, six genes (*RBPMS2*, *PDK4*, *IGK*, *SBSN*, *IFIT3* and *HSPB6*) were likely to be the biomarkers of tumorigenesis for esophageal squamous carcinoma due to the fact that the biological processes in which they participate are closely related with the development of esophageal squamous carcinoma. Statistical analysis indicates that effectiveness of the detected biomarkers of esophageal squamous carcinoma. The proposed method could be extended to other complex diseases for detecting the molecular features of pathopoiesis and targets for targeted therapy.

## 1. Introduction

Esophageal cancer is one of the leading causes of cancer-related deaths in the world [1]. Esophageal cancer has a poor prognosis and high mortality rate, with an estimated 16,910 new cases and 15,910 deaths projected in 2016 in the United States [2]. Furthermore, the survival rate for affected patients is very low at later stages, ranging from 10% in Europe to 16% in the United States [1]. Due to the limitations of the technologies of diagnosis and treatment, it is difficult to find an effective method for the diagnosis and therapy for the esophageal cancer.

There are various techniques in tissular or cellular levels for the identification and classification of esophageal cancer, including X-ray examination, positron emission tomography, magnetic resonance imaging and biomarker testing such as prostate-specific antigens in the blood circulation [3]. However, there are several deficiencies in adequately describing the changes between esophageal cancer and normal in cellular levels. The important genetic changes always lead to specific changes in molecular. With the development of personalized genome sequencing and the use of varieties of omics data, it is available to find the molecular features of tumorigenesis or targeted therapy targets by using the data mining method to process the transcriptome data [4], epitranscriptome data [5], metabolome data [6], etc.

A systematic review has come to a conclusion that gene expression profiles could be used to predict clinical outcome and to select optimal personalized therapy [7]. In previous studies, many cancer-related genes have been identified based on gene transcriptome data. Some of these genes have been confirmed as tumor suppressor genes, and some have been treated as targets in cancer targeted therapy [8,9,10]. However, these studies mainly focus on identifying differentially expressed genes, but neglect the topological difference between tumor and normal networks.

In this work, we proposed a method to select candidate biomarkers of esophageal squamous carcinoma based on the topological differences between the gene-gene interaction networks for esophageal squamous carcinoma and normal cells. Specifically, the gene-gene interaction networks were established based on spearman rank correlation coefficients among genes in esophageal squamous carcinoma and normal, respectively. Then, the topological difference was analyzed between these two networks. Experimental analysis has demonstrated the effectiveness of the detected results.

## 2. Results

**Figure 1:**
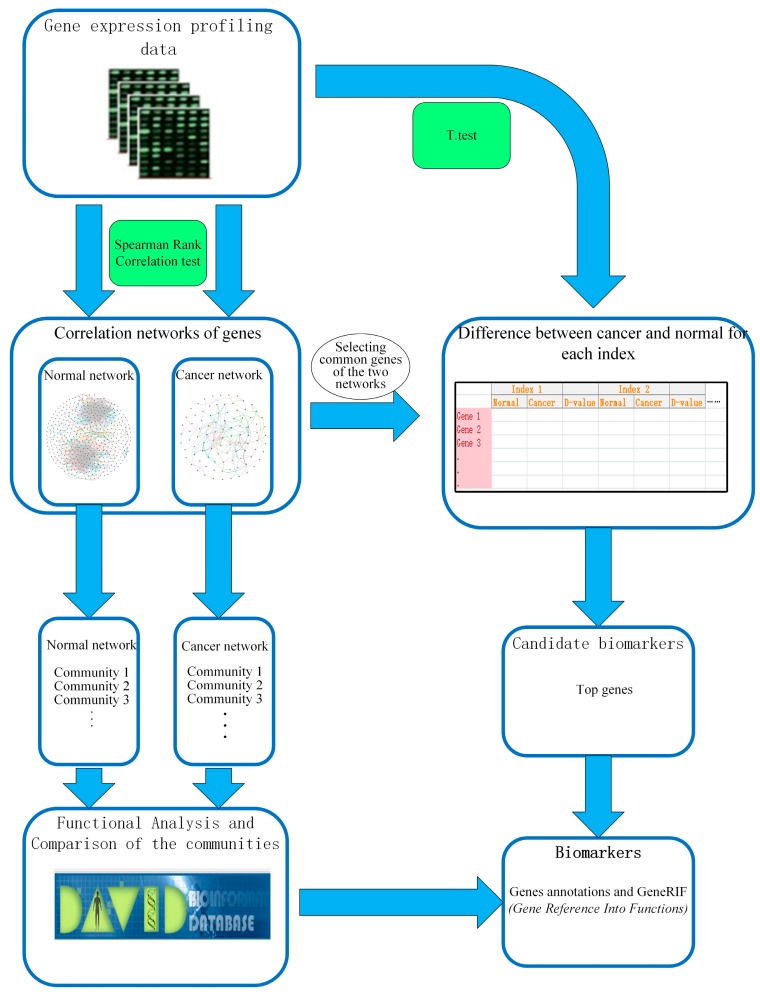
The overall framework for identification of biomarkers.

In our work, we established the gene-gene interaction networks for esophageal squamous carcinoma and normal. In addition, the way to detect candidate biomarkers was applied on these networks. The overall framework of our work is shown in Figure 1.

### 2.1. Gene Networks

In our work, we calculated the Spearman rank correlation coefficients of all gene pairs in the cancer group and the normal group, respectively [11]. Further, 0.8 was chosen as a threshold for filtering relationships between genes based on the distribution of correlation coefficients. In other words, if the correlation coefficient of a gene pair was greater than 0.8, then an edge was established between the two genes; otherwise, no edges were established. In addition, a statistical analysis was implemented to verify the reliability of the selected relationships. To be specific, we calculated the repetition rate of each relationship for both esophageal cancer and normal cells. We found that the average repetition rate of relationships between genes in normal cells was 0.69 and that in esophageal cancer cells was 0.72. It implies that most relationships between genes hardly depend on the selected samples [12]. Here, the gene-gene interaction network for esophageal cancer was termed as the cancer network, while that for normal was named as the normal network.

**Figure 2:**
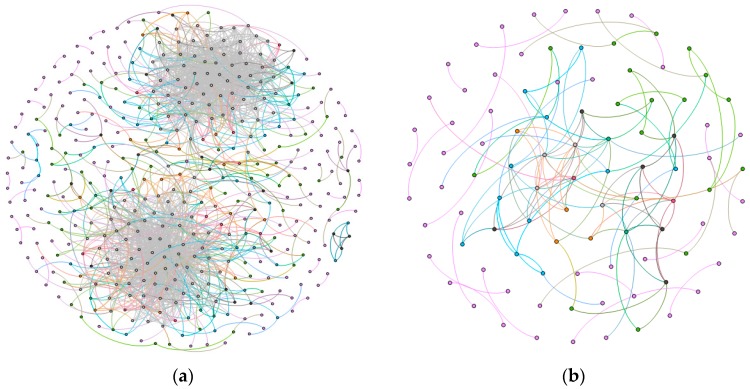
The gene-gene interaction networks, (**a**) is the normal network; and (**b**) denotes the esophageal squamous carcinoma network.

As shown in Figure 2, the normal network had 441 nodes and 2047 edges, while the cancer network contained 95 nodes and 140 edges. The average degree and the diameter of the normal network were respectively 4.65 and 9, while 1.24 and 4 in the cancer network. Moreover, the average clustering coefficients of them were 0.17 and 0.14. According to Figure 2, we could see that the number of nodes and edges in normal network was much larger than those in cancer network. The main reason to illustrate the fact is that in esophageal cancer cells, some of the normal pathways will be blocked, which may lead to the phenomenon of dedifferentiation in the process of cancer cell formation [13].

#### 2.1.1. Comparison of the Communities between Esophageal Squamous Carcinoma and Normal

In what follows, we respectively detected communities in the cancer and normal networks and then analyzed the biological processes in which the genes in a community mainly participated. In the normal network, we detected two communities, i.e., Community 1 and Community 2. According to Table 1, we found that the genes in Community 1 were mainly participated in the extracellular matrix, cell framework, and cell membrane structure.

It also can be seen that the genes in Community 2 were mainly involved in cell and external signal transduction and protein synthesis. Previous literature indicated that the extracellular matrix provides a suitable environment for cells and maintains its survival and activity, while the signal transduction system affects the shape of cells, metabolism, function, migration, proliferation and differentiation. It is suggested that the genes involved in these two communities maintain the operation of normal cells [14]. However, these two communities did not appear in the cancer network, which suggests that the incidence of esophageal cancer is associated with the loss of regulation of the gene associated with extracellular matrix, as well as cell and external signal transduction [15]. It is indicated that due to the dysfunction of above genes, cancer cells are characterized by infinite proliferation, mitochondrial polymorphism, swelling, hyperplasia, cytoskeletal disorders, abnormal skeletal assemblage and changes in cell surface characteristics in esophageal cancer tissues.

Furthermore, we selected the two largest communities (Community A and Community B) in the cancer network for the functional enrichment analysis. The main functions of these are shown in Table 2. According to Table 2, the genes in Community A were mainly involved in cell adhesion and immunoglobulin functions. Previous research has shown that the normal cells are connected in three ways: adherens junction, tight junction and gap junction [16]. Adherens junction relies on adhesion such as cadherin, actin and catenin to achieve intercellular adhesion. The other two connections are mainly used as intercellular channels for the transport of nutrients and signals. The cancer cells are easily invaded and transferred due to the fact that the intercellular adhesion of cancer cells compared to normal cells in the same tissue has a significant reduction [17]. It indicates that the interactions between genes in Community A inhibit the adhesion of adherent cells. It was also found that genes of immunoglobulins cannot express in normal tissue cells but only express in immune cells. Many studies have shown that a variety of cancer cells can express immunoglobulins which play a role as the growth factor, which suggests that cancer cells cannot grow and survive without immunoglobulin. Moreover, the purified immunoglobulins isolated from human cancer tissue also inhibit lymphocyte proliferation [18]. Immunoglobulins derived from cancer cells have the dual effects of promoting the growth of cancer cells and inhibiting the cellular immune function of the host cells.

In addition, as can be seen from Table 2, the genes in Community B were mainly involved in the following biological functions: extracellular matrix, extracellular secretion and collagen. Compared with the normal network, the number of functional types enriched in the extracellular matrix was larger than that in the cancer network. It indicates that the impact of genes on the extracellular matrix are distinct between esophageal squamous carcinoma and normal cells, which affects the composition of the extracellular matrix, the surrounding environment of cancer cells, and makes that more conducive to its proliferation and survival [19]. Thus, esophageal cancer cells may regulate the extracellular matrix environment by extracellular secretion to make them more conducive to the formation, development, and proliferation [20].

#### 2.1.2. Differential Analysis Based on Global Centrality Indexes

The global centrality indexes used in this work were degree, eigenvector centrality and core [21]. We calculated three global centrality indexes of genes. The genes which showed large differences between the cancer network and normal network were listed in Table 3. The top ten genes with a large difference of degree and high significance (*p*-value < 0.02, and FDR < 0.1) were selected into further consideration. We found that five of them were associated with cancer, namely *BNIPL*, *PRKG1*, *ABI3BP*, *MIR145*, and *ERBB3*. To be specific, *BNIPL* inhibits cell growth through cell cycle and apoptosis, and it could induce the occurrence of cancer [22,23]. *PRKG1* improves the activity and invasion of cancer cells, and it is also indicated that *PRKG1* plays a role as an intermediary in the epidermal growth factor receptor (EGFR)-mediated cell death, likely via apoptotic pathway [24]. *ABI3BP* plays an important role in the proliferation of replicative senescence and may serve as a trigger of tumor development [25]. Low *MIR145* expression levels in conjunction with elevated *SIP1* expression levels may contribute to cancer development [26] and might carry crucial roles in laryngeal squamous cell carcinoma tumorigenesis, prognosis, metastasis, chemoresistance, and recurrence through regulating stem cell properties of tumor cells [27]. *ERBB3* is proto-oncogene and promotes differentiation of undifferentiated cancer cells and plays an important role in cancer formation and are related to a favorable prognosis [28,29].

We also detected the top 10 genes with a large difference of eigenvector centrality and *p*-value < 0.02 with FDR < 0.1 (*t*-test). It was found that among the 10 genes, seven genes (*SORBS1*, *PGM5*, *COL3A1*, *MYLK*, *MIR100HG*, *RBPMS2* and *MIR145*) were confirmed to be related to cancer by analyzing the biological function of these genes. Specifically, *SORBS1* and *PGM5* were proposed to be involved in the assembly of myocytes and myofibrils. In addition, it was found that myofibrillar cells are a class of cells with low level of differentiation in myofibroblasts, which are an important source of matrix remodeling protein in the tumor microenvironment and participates in tumor angiogenesis [30]. *COL3A1* promotes the metastasis and invasion of cancer cells [31]. *MYLK* is responsible for the high proliferative ability of cancer cells through anti-apoptosis in which the *p38* pathway is involved and represents a mediator of invasive behavior of cancer cells that are regulated by the ZEB1/miR-200 feedback loop [32,33]. It has been reported that *MIR100HG* has been used as proto-oncogene and *RBPMS2* is used as a target for gastric cancer markers and cancer targeted therapy [34].

Core score measures the degree to which a node belongs to the core in the core-periphery structure of a network [35]. The nodes with large core scores were referred to core nodes, while those with small core scores were in terms as periphery nodes. Core nodes in a network might play a different role from periphery ones [36]. Hence, core nodes might be more influential or powerful than periphery ones. We calculated the difference of each gene’s core score between the cancer and the normal networks. The first ten genes with large absolute difference value and *p*-value < 0.02 with FDR < 0.1 were selected for further analysis. We found that five genes were related to cancer, including *BNIPL*, *ERBB3* and *PRKG1*, which were also been detected by degree or eigenvector measures. The other two genes are *PDK4* and *YOD1*. It has been reported that the upregulation of *PDK4* makes intracellular glucose metabolism pathways prone to glycolysis to promote cell proliferation [37]. Besides, *PDK4* promotes the occurrence of cancer. An important feature of cancer cells is that the aerobic respiration is replaced by glycolysis, which is closely related to the occurrence and development of cancer [38].

#### 2.1.3. Differential Analysis Based on Local Centrality Indexes

Above global indexes could reflect the centrality of a node in the whole network. However, these indexes may not reflect its importance in a local subnetwork. For example, if the degree of a node is five, the importance and influence of the node will be different in terms of the different size of networks (e.g., a network with 10 nodes and that with 100 nodes). In what follows, we defined two indexes (local mean degree difference and local eigenvector centrality difference) to measure the difference of the same node in different networks.

We identified the community structures in the two simplified networks [39]. Further, the local mean degree difference and local eigenvector centrality difference of each node were calculated. As shown in Table 4, we selected the first 15 genes with a large difference of local centrality indexes for further analysis. The 15 genes were taken into statistical analysis (*t*-test). It was found that the *p*-values of the 15 genes were smaller than 0.02.

Nine of the 15 genes were suggested to be related to esophageal cancer, where six of them were newly detected, and other three were previously described. It is reported that *SPINK7* is a novel candidate of the tumor suppressor gene identified from human esophagus and plays an important role in the carcinogenesis of esophageal cancer [40]. Many studies suggested that *SPINK7* may function as a tumor suppressor gene regulating the protease cascades during carcinogenesis and invasion of esophageal cancer [41,42,43]. *IGK* is an immunoglobulin variable kappa gene involved in the immune function to cancer cells. The findings provided *IGK* as a novel diagnostic marker for risk stratification in human cancer [44]. It may be used as a new marker for the diagnosis of esophageal cancer. It has been reported that *SBSN* can be used as a new candidate proto-oncogene and has a potential relationship with the angiogenesis of cancer [45]. Besides, previous studies suggested that suprabasin (*SBSN*) plays an important oncogenic role in promoting proliferation and tumorigenesis of esophageal squamous cell carcinoma [46]. It has been reported that *IFIT3* is a proto-oncogene, and its upregulation can maintain the cellular condition of pseudo inflammatory in cancer tissue [47]. Studies have shown that inflammation plays an important role in cancer formation and growth, because it can promote the formation of cancer and activate some transcription factors such as Angiogenesis regulator, proliferation medium and anti-apoptotic factors. *HSPB6* can inhibit the growth of hepatocellular carcinoma by inhibiting the *AKT* pathway, and in other cancers, it is also found to inhibit the growth of cancer and can be used as a new marker for cancer diagnosis [48]. *RSPO3* has been reported as a regulatory gene in the cancer tissue as a cyclical gene [49,50].

Similarly, we analyzed the results of the local eigenvector centrality. Among the top 15 genes, nine genes were proposed to be related to cancer in previous literature. What is more, except *SCEL* and *IGDCC4*, the other seven genes were also detected by other centralities indexes. That is, as an esophageal cancer related gene, *SCEL* can only be identified by local eigenvector centrality [51]. It implied the effectiveness of the way to identify cancer related molecular by local eigenvector centrality. A pervious study has identified *IGDCC4* as a novel oncofetal surface marker for murine and human *HCC* and it is specifically expressed by epithelial tumor cells but not in preneoplastic stages and is a promising marker [52].

According to Table 3 and Table 4, among the top 10 or 15 genes selected for each index, five genes detected by degree are related to esophageal cancer and seven genes by eigenvector centrality; five genes are identified by core score and nine genes by both local mean degree and local eigenvector centrality. At last, we integrated all of the above genes related to esophageal cancer and identified 21 non-redundant genes that were related to esophageal cancer, among which seven genes have been confirmed to be biomarkers of esophageal cancer by previous research, and six genes (*RBPMS2*, *PDK4*, *IGK*, *SBSN*, *IFIT3* and *HSPB6*) may be novel biomarkers for the diagnosis of esophageal cancer.

#### 2.1.4. Performance Comparison

In this paper, a network-based method (i.e., topological parameters analysis) was chosen to compare with the proposed method. The differentially expressed genes and the network were obtained by use of the Cytoscape software (3.5.1, National Institute of General Medical Sciences (NIGMS), Bethesda, MD, USA) [53]. Protein pairs whose combined score were larger than 0.4 were assembled for network construction by Cytoscape software [54]. Then, the values of degree and betweenness of each node were calculated. The nodes of which the values of degree were larger than 5 and the values of betweenness were larger than 0.03 were selected as hub-bottleneck genes [55]. The top six nodes are all related to cancer, but there are few reports of their association with esophageal cancer (Table 5). The proposed method was superior to the comparison method, since the proposed method found a larger number of genes which were reported to be related to esophageal cancer in previous research.

## 3. Discussion

In this work, we detected the tumorigenesis-related genes for esophageal squamous carcinoma based on topological differential analysis and explored the functional divergence between esophageal squamous carcinoma and normal. To be specific, five indexes were selected to reflect the global and local differences between cancer and normal networks: the degree of nodes, eigenvector centrality, the core score in core–periphery structure, the local mean degree and the local eigenvector centrality. We calculated the difference value of above indexes of each gene between the normal and cancer networks, and found 21 genes which may play important parts in the pathogenesis of esophageal squamous carcinoma. Among the detected 21 genes related to esophageal squamous, nine of 13 carcinoma, seven genes have been confirmed to be related to esophageal squamous carcinoma by previous research and the other 14 genes are newly detected biomarker candidates for esophageal squamous carcinoma. In addition, six of the newly detected genes are closely related to the formation of esophageal cancer, i.e., *RBPMS2*, *PDK4*, *IGK*, *SBSN*, *IFIT3* and *HSPB6*. Previous works have proved that degree and betweenness are useful to find hubs and bottlenecks which are supposed to be essential to certain biological function. In this work, we found that the hub-bottlenecks which were detected based on degree and betweenness showed little relation to esophageal squamous carcinoma. Therefore, the bottlenecks may be not suitable for the markers to the difference of network structures.

However, as there is a lack of knowledge about genes, there is not enough evidence for the rest of the genes selected to have specific relationships to esophageal squamous carcinoma, and the genes determined to be the biomarkers of esophageal cancer are worthy of further study. More generally, the current method could be extended to other complex diseases for detecting the molecular features of pathopoiesis and targets for targeted therapy.

## 4. Methods

### 4.1. Data Source and Data Processing

We used the specimens of GSE23400 (a Gene Expression Omnibus accession number for microarray data), obtained from National Center for Biotechnology Information (NCBI, http://www.ncbi.nlm.nih.gov/). These specimens contained the expression data of 22,645 probes in 102 specimens, where 51 were the samples of esophageal squamous carcinoma and the rest 51 were the normal samples. The microarray expression data was normalized through the Microarray Suite 5 (Mas5 for short) algorithm [56]. The Mas5 algorithm generated a *p*-value and a mean signal value that assessed the reliability of the expression level for each probe. If the mean value of the *p*-value of each sample for a probe was larger than 0.02, then the probe data was filtered out. Besides, the probes matched no gene symbols were also deleted. If more than one probe corresponded to a gene, then we calculated the mean value of these probes in a sample to get the expression data of the gene. Finally, the expression data of 6047 genes were obtained.

### 4.2. Spearman Rank Correlation Coefficient

Spearman rank correlation coefficient of vectors *X* and *Y* reflects how closely *X* and *Y* are related by monotonic functions. The vectors *X* and *Y* are first respectively ranked according to the elements in them to obtain the vectors P and Q. Then, we calculate the Spearman rank correlation coefficient ρ of vectors *X* and *Y* as Equation (1):

(1) $$\rho \left(X,\;Y\right)=1-\frac{6\sum_{i=1}^{n}{\left(p_{i}-q_{i}\right)}^{2}}{n\left(n^{2}-1\right)},$$

where *n* is the dimension of *X*, and $p_{i}$ and $q_{i}$ are the *i*th entry of P and Q, respectively.

### 4.3. Centrality Measures

For a given network G = (V, E), V represents the set of vertices and E is the set of edges. Let $A=a_{v,t}$ be the adjacency matrix. In what follows, the centrality measures are presented.

***Degree centrality*.** Degree used to describe the number of links connected to a node. The degree of a node i is calculated as Equation (2):

(2) $$k_{i}=\underset{j\in N}{\sum }a_{ij}$$

where $a_{ij}$ is the element in the *i*th row and *j*th column of *A*.

***Eigenvector centrality.*** The eigenvector centrality score of node *v* is calculated as Equation (3).

(3) $$x_{v}=\frac{1}{\gamma }\underset{t\in M\left(v\right)}{\sum }x_{t}=\frac{1}{\gamma }\underset{t\in G}{\sum }a_{v,t}x_{t}$$

where *M*(*v*) is a set of the neighbors of *v*, and γ is a constant.

***Core score.*** Suppose *C* is a matrix with the same dimension to the adjacency matrix *A*. The core quality was calculated as Equation (4) [21].

(4) $$R_{\gamma }=\underset{i,j}{\sum }A_{ij}C_{ij},$$

where *g* presents the core quality, the elements $C_{ij}$ of the core matrix are given by $C_{ij}=C_{i}\times C_{j}$ and $C_{i}\geq 0$ is the local core value of the ith node.

We select a core vector *C* that maximizes $R_{\gamma }$ and is a normalized (so that its entries sum to 1) shuffle of the vector $C^{*}$ whose components $C_{i}^{*}\;=\;g\left(i\right)$ are determined by a transition function *g*.

(5) $$C_{i}^{*}\left(\alpha ,\beta \right)=g_{\alpha ,\beta }\left(i\right)=\{\begin{array}{c} \frac{i\left(1-\alpha \right)}{2\lfloor \beta N\rfloor },\;i\in \left(1,\ldots ,\lfloor \beta N\rfloor \right)\;\\ \frac{\left(i-\lfloor \beta N\rfloor \right)\left(1-\alpha \right)}{2\left(N-\lfloor \beta N\rfloor \right)}+\frac{1+\alpha }{2},\;i\in \left(\lfloor \beta N\rfloor +1,\ldots ,n\right) \end{array},\;\alpha ,\beta \in \left[0,\;1\right].$$

When *β* = 0, only the top case in Equation (5) applies; when *β* = 1, only the bottom case applies. The parameter *β* sets the size of the core: as *β* varies from 0 to 1, the number of nodes included in the core varies from *N* to 0. The parameter *α* sets the size of the score which jumps between the highest-scoring peripheral node and the lowest-scoring core node. With the transition function Equation (5) and the product *C* form Equation (4) for the core–matrix elements, the core quality is given by

(6) $$R_{\gamma }=R_{\alpha ,\beta }=\underset{i,j}{\sum }A_{ij}C_{ij}=\underset{i,j}{\sum }A_{ij}C_{i}\times C_{j}$$

The core score of each node *i* is defined as

(7) $$CS\left(i\right)=Z\underset{\gamma }{\sum }C_{i}\left(\gamma \right)\times R_{\gamma }$$

where the normalization factor *Z* is chosen so that $max_{k}\left[CS\left(k\right)\right]=1$.

***Local eigenvector centrality.*** The local eigenvector centrality score of node *i* can be defined based on eigenvector centrality scores of nodes as:

(8) $$x_{i}=\frac{1}{\gamma \prime }\underset{ti\in Mi\left(i\right)}{\sum }x_{ti}=\frac{1}{\gamma \prime }\underset{ti\in g}{\sum }a_{i,ti}x_{ti}$$

where Mi(i) is a set of the neighbors of *i* and γ′ is a constant.

***Local mean degree.*** The local mean degree of a node is the ratio of its degree of in the community to the sum of all the degrees of the community’s nodes. The local mean degree $d_{v\left(i\right)}$ is defined as:

(9) $$d_{v\left(i\right)}=\frac{d_{v}}{n_{i}}$$

where $d_{v}$ is the degree of node i in the community, and the sum of all nodes in the community is $n_{i}$.

## Figures and Tables

**Table 1:** Function enrichment of genes in communities of the normal network.

Community 1			Community 2		
Cluster	Term	*p*-Value	Cluster	Term	*p*-Value
1	Extracellular matrix	9.52 × 10^−6^	1	Notch signaling pathway	4.00 × 10^−4^
	Secreted	0.0013		Epidermal growth factor (EGF)-like calcium-binding	0.0315
	Extracellular region	0.01416		conserved site	0.0316
2	Muscle contraction	1.60 × 10^−7^		EGF-type asparagine hydroxylation site	0.0340
	Actin-binding	0.0153		EGF-like, conserved site	0.0456
	Cytoskeleton	0.0386		EGF_CA	0.0469
3	Cell membrane	8.50 × 10^−4^	2	Golgi apparatus	0.0229
	Membrane	0.0058			
	Plasma membrane	0.0026			

**Table 2:** Function enrichment of genes in communities of the cancer network.

	Community A			Community B	
Cluster	Term	*p*-Value	Cluster	Term	*p*-Value
1	muscle contraction	1.07 × 10^−6^	1	proteinaceous extracellular matrix	2.95 × 10^−5^
	stress fiber	7.67 × 10^−4^		extracellular matrix	4.34 × 10^−5^
	focal adhesion	0.0029		extracellular region	0.0226
	cytoskeleton	0.0388		extracellular matrix	3.53 × 10^−7^
	cell junction	0.0753		secreted	7.31 × 10^−5^
2	immunoglobulin I-set	0.0049		signal peptide	3.45 × 10^−4^
				signal	0.0013
				compositionally biased region: Cysrich	0.004
			2	collagen catabolic process	6.22 × 10^−6^
				extracellular matrix	4.34 × 10^−5^
				collagen fibril organization	2.34 × 10^−4^
				extracellular matrix organization	0.0057
				endoplasmic reticulum lumen	0.0079
				extracellular region	0.0226
				extracellular matrix	3.53 × 10^−7^
				Ehlers–Danlos syndrome	3.34 × 10^−5^
				collagen triple helix repeat	0.0010
				hydroxylation	0.0015
				collagen	0.0016

**Table 3:** TOP 10 genes based on global centrality indexes.

Gene	Degree	*p*-Value	Gene	Eigenvector	*p*-Value	Gene	Core Score	*p*-Value
*C1orf116*	61	1.6 × 10^−14^	*SORBS1*	1	1.7 × 10^−5^	*C1orf116*	0.50	1.6 × 10^−14^
*NEXN*	48	0.0145	*COL3A1*	0.93	1.5 × 10^−11^	*BNIPL*	0.40	3.8 × 10^−15^
*BNIPL*	45	3.8 × 10^−15^	*MYLK*	0.90	0.0057	*PRSS27*	0.38	4.0 × 10^−12^
*ERBB3*	44	7.8 × 10^−16^	*PGM5*	0.87	7.4 × 10^−7^	*ERBB3*	0.34	7.8 × 10^−16^
*SCN7A*	43	0.000108	*MIR100HG*	0.68	0.0003	*CNFN*	0.33	4.4 × 10^−17^
*PRSS27*	40	4.0 × 10^−12^	*RBPMS2*	0.60	0.0002	*PRKG1*	0.28	0.01496
*MRGPRF*	37	0.0002	*SCN7A*	0.58	0.0001	*PDK4*	0.26	1.6 × 10^−5^
*PRKG1*	37	0.0149	*C1orf116*	0.56	1.6 × 10^−14^	*CCDC64B*	0.26	2.7 × 10^−14^
*ABI3BP*	36	3.5 × 10^−5^	*MIR145*	0.56	0.0003	*YOD1*	0.24	4.6 × 10^−12^
*MIR145HG*	33	0.0003	*CCDC64B*	0.50	2.7 × 10^−14^	*METRNL*	0.24	3.7 × 10^−10^

**Table 4:** Top 15 genes based on local centrality indexes.

Gene	Local Degree	*p*-Value	Gene	Local Eigenvector	*p*-Value
*FAM3D*	0.63	5.2 × 10^−13^	*SBSN*	0.95	1.2 × 10^−16^
*SBSN*	0.58	4.7 × 10^−5^	*OGN*	0.95	5.2 × 10^−13^
*SPINK7*	0.56	7.9 × 10^−6^	*IFIT3*	0.93	1.7 × 10^−14^
*HSPB6*	0.55	1.6 × 10^−5^	*PDK4*	0.90	8.4 × 10^−6^
*LINC01279*	0.50	0.1191	*RSPO3*	0.89	0.003063
*SCEL*	0.47	3.5 × 10^−5^	*ABI3BP*	0.87	6.2 × 10^−13^
*SMIM5*	0.47	8.4 × 10^−6^	*HSPB6*	0.84	5.8 × 10^−17^
*OGN*	0.47	1.2 × 10^−16^	*FAM3D*	0.75	4.7 × 10^−5^
*YOD1*	0.47	1.7 × 10^−14^	*SPINK7*	0.72	4.6 × 10^−12^
*PELI1*	0.46	1.7 × 10^−5^	*SORBS1*	0.70	6.48 × 10^−8^
*GNG2*	0.44	0.00306	*LINC01279*	0.70	0.00236
*IFIT3*	0.43	6.4 × 10^−8^	*PELI1*	0.70	7.92 × 10^−6^
*IGDCC4*	0.43	0.00236	*GNG2*	0.70	0.091054
*IGK*	0.43	0.09105	*IGK*	0.70	3.76 × 10^−5^
*RSAD2*	0.43	3.7 × 10^−5^	*RSAD2*	0.68	3.56 × 10^−5^

**Table 5:** The hub-bottleneck nodes obtained by the comparison method.

R	Gene	Description	Degree	BC
1	*ACTA2*	actin, alpha 2, smooth muscle, aorta	8	0.45
2	*PRKG1*	protein kinase, cGMP-dependent, type I	7	0.40
3	*GNB1*	G protein subunit beta 1	7	0.05
4	*COL1A2*	collagen type I alpha 2 chain	6	0.18
5	*ITGA1*	integrin subunit alpha 1	6	0.06
6	*MYH11*	myosin heavy chain 11	6	0.04
7	*COL3A1*	collagen type III alpha 1 chain	5	0.15
